# Case report: Disseminated pulmonary mucormycosis involving spleen in diabetic patient with aggressive surgical approach

**DOI:** 10.1016/j.ijscr.2018.11.057

**Published:** 2018-11-29

**Authors:** Saleh Alqhamdi, Bandar Idress, Ashwag Alharbi, Nawaf Aljurais

**Affiliations:** Department of General Surgery, Prince Sultan Military Medical City, Riyadh, Saudi Arabia

**Keywords:** IDDM, Insulin-Dependent Diabetes Mellitus, CT, computed tomography, HSCT, hematopoieticstem cell transplant recipients, SOT, solid organ transplant, BAL, Broncho alveolar lavage, Disseminated mucormycosis, Pulmonary mucormycosis, Diabetic

## Abstract

**Introduction:**

Mucormycosis is a life threatening fungal infection that occurs in immunocompromised patients.

**Presentation of case:**

A 36-year-old Saudi male with known case of Insulin-Dependent Diabetes Mellitus (IDDM), who was presented with productive cough and diarrhea. Computed tomography (CT) revealed a disseminated fungal invasion of the lungs and spleen with invading the fundus of the stomach. An aggressive surgical approach and amphotericin B treatment resolved his condition.

**Discussion:**

Disseminated Mucormycosis in one organ can spread hematogenously to other organs and can cause severe morbidity and mortality in high risk individuals. The diagnosis of mucormycosis is usually delayed, Antifungal therapy alone is insufficient for mucormycosis, and surgical debridement for all infected tissue is often required.

**Conclusion:**

Mucormycosis is a life threating infection, and usually the diagnosis late because of non-specific clinical, radiological feature and need biopsy for confirmation of the organism. The cornerstone for the management and increase the survival rate remain on rapid diagnosis, treatment of the underlying predisposing condition, and urgent surgical debridement.

## Highlights

• Mucormycosis is life threating infection.
• Disseminated mucormycosis is a rare condition with high mortality rate .
• The cornerstone for the management its remain on rapid diagnosis, treatment of the underlying predisposing condition, and urgent surgical debridement.

## 1 Introduction

Mucormycosis is an emerging angioinvasive infection caused by saprophytic aerobic fungus, that belongs to a group of fungi called Mucoromycotina in the order Mucorales.

Mucormycosis was 1st described by Paulltaf in 1885 [1], has emerged as the third most common invasive mycosis in order of importance after candidiasis and aspergillosis in patients with hematological and allogeneic stem cell transplantation [2]. Which can cause rhino-cerebral, pulmonary, gastro-intestinal, cutaneous or disseminated disease. Mucormycosis is the second most frequent mould infection seen in immunocompromised individuals which has a very high mortality rate reaching 50–80% even with treatment. The commonest organism’s which cause human infection are *Rhizopus oryzae* (R. arrhizus) and *Rhizopus microsporus*. In tissue as well as in culture, these moulds form characteristic broad, non-septate or sparsely septate hyphae with right-angled branching.

The major risk factors predisposing individuals to the disease include prolonged or profound neutropenia, uncontrolled diabetic and metabolic acidosis, malnutrition, use of corticosteroids and treatment with the iron-chelating agent such as desferrioxamine. High-risk patient groups include hematopoieticstem cell transplant (HSCT) recipients, solid organ transplant (SOT) recipients, patients with hematological malignancies, patients with type I or II diabetes mellitus, burns patients, injection drug users and individuals with no apparent immunological defects [3].

Mucormycosis infections occur following in halation, implantation, or ingestion of spores. The Mucormycosis infections are characterized by extensive angioinvasion that results in vessel thrombosis and subsequent tissue necrosis. Ischemic necrosis of infected tissues can prevent delivery of leukocytes and antifungal agents to the foci of infection. This angioinvasion likely contributes to the capacity of the organism to hematogenously disseminate to other target organs [4].

Because mucormycosis is such an aggressive infection, an early diagnosis is essential for the successful management. The microscopic demonstration of Mucorales in clinical material taken from necrotic lesions or in sputum or Broncho alveolar lavage (BAL) fluid is more significant than their isolation in culture. If the management of mucormycosis is to be successful, treatment should be initiated as soon as the diagnosis is suspected, the underlying metabolic or immunological disorders that precipitated the infection must be corrected, infected necrotic tissue must be removed and an appropriate antifungal agent must be administered.

Immunosuppressive and myelotoxic drugs should be reduced in dose or discontinued, provided this will not harm the patient. Iron chelation treatment with desferrioxamine should also be discontinued. Amphotericin B remains the most potent drug available for mucormycosis [3].

## 2 Case report

The work has been reported in line with the SCARE criteria [30].

In November 2017, A 36 years old Saudi male with known case of IDDM, presented to the emergency department complained of 1 month history of diarrhea and cough. Diarrhea was watery with productive cough, yellowish in color, associated with shortness of breath and weight loss, no history of hemoptysis or abdominal pain, no contact with sick patient or using drug. No past surgical history. No significant family neither psychological history.

On examination: Conscious oriented alert, not on respiratory distress, not pallor neither cyanosis, with lower limb edema grade 3, the patient was visibly underweight. Cardiovascular examination was unremarkable, Chest examination decrease air entry on left side with inspiratory crackle. Other systemic examinations were unremarkable.

**Fig. 1:**
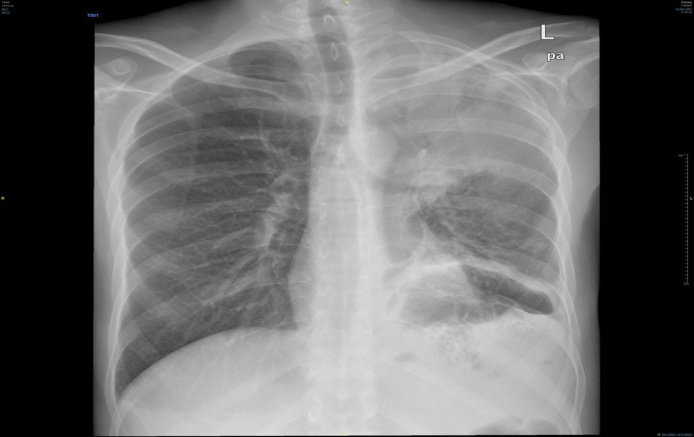
Chest x-ray reveled cavity at left side with pleural effusion.

**Fig. 2:**
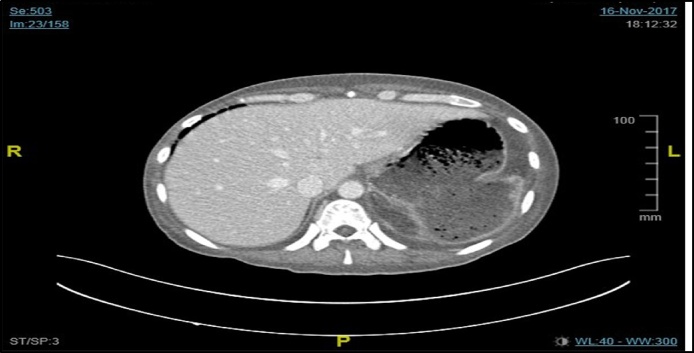
CT chest axial view showing splenic abscess invading the stomach.

**Fig. 3:**
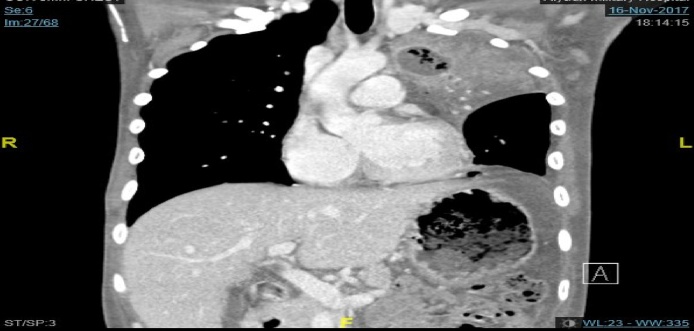
CT chest coronal view showing left upper lobe consolidation and cavitation.

**Fig. 4:**
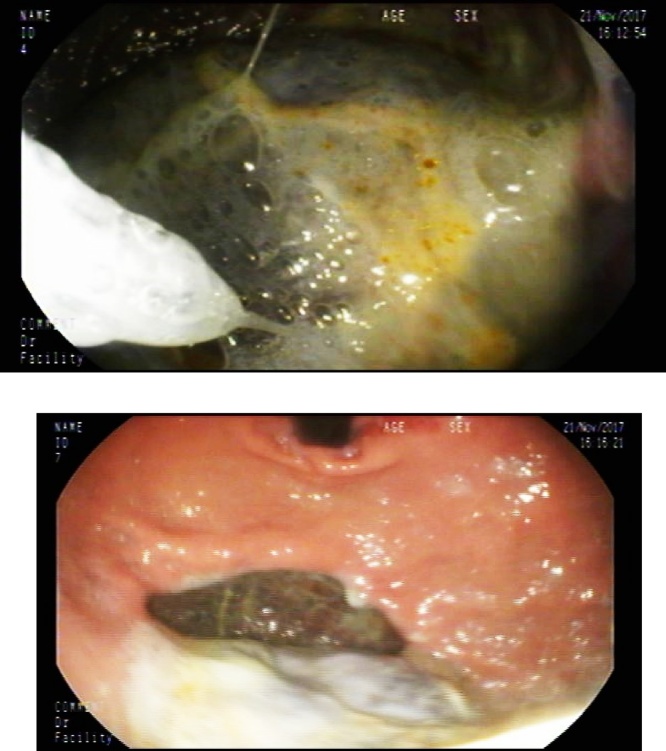
Endoscopy showing spleen invading the fundus of stomach.

His workup WBC 14.2 × 10^9^/L,hgb7.1 g/dl, platelets 660 × 10^9^/L, albumin 18 g/L, ESR 89 mm/h, CRP 74 mg/L and ECG was showing normal sinus rhythm. Chest x-ray revealed a cavity at the left side with pleural effusion (Fig. 1). Patient was admitted for workup for his chronic diarrhea. His CT of chest and abdomen (Fig. 2, Fig. 3) revealed left upper lobe air space consolidation associated with secretion with in left upper main bronchus as well as cavity lesion inside, measuring 3 × 4 × 3 cm, with bilateral plural effusion, abdominal wise there was left inferior subpleural cavity like abscess measuring about 11 × 10 × 12 cm invading pleural and splenic communicating with posterior fundus of the stomach, with upper pole splenic infarction. Bronchoscopy of the left bronchus was having thick mucus in which BAL and biopsy was taken, the BAL culture and sensitivity was negative, while the biopsy was positive for mucormycosis. Upper GI endoscopy showed gross spleen invading fundus of the stomach as shown in (Fig. 4).

**Fig. 5:**
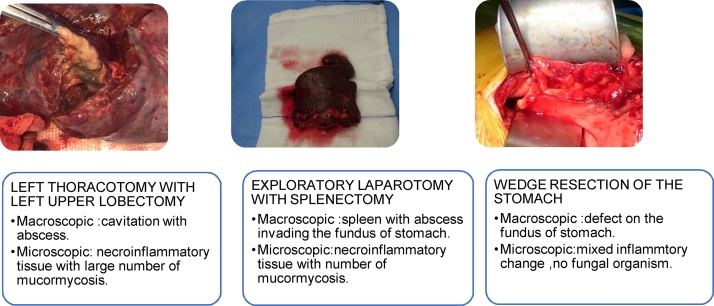
Surgical management.

Surgical management was considered for him including: left thoracotomy with left upper lobectomy, exploratory laparotomy, splenectomy and wedge resection of the stomach (Fig. 5).

**Fig. 6:**
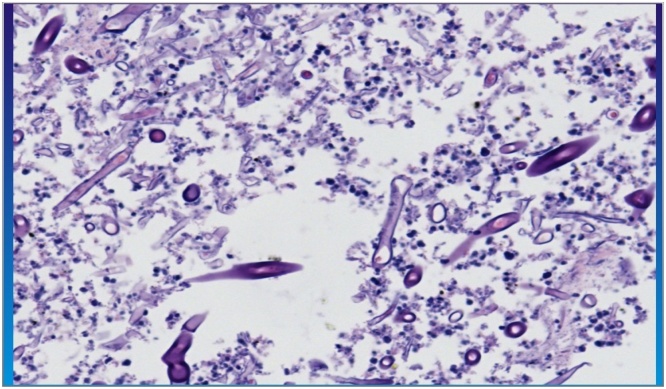
Necroinflammatory tissue with large number of fungal hyphae consistent with mucormycosis.

He was covered pre- and post-surgery with amphotericin B and micafungin for 2week. Culture of specimens from lung and spleen showed Mucormycosis as shown in (Fig. 6). After two weeks patient was discharged in good condition, for follow up at outpatient clinic.

## 3 Discussion

Disseminated mucormycosis is a rare condition and the most common reported sites for this condition are; sinuses (39%), lungs (24%), and skin (19%). Dissemination developed in 23% of these cases. Although brain is a common site to spread the metastatic lesions may also be found in the liver, spleen, heart, and other organs. The overall mortality rate for the disease is 44% in diabetics, 35% in patients with no underlying conditions and 66% in patients with malignancies [15].

Disseminated Mucormycosis in one organ can spread hematogenously to other organs and can cause severe morbidity and mortality in high risk individuals. Our patient was having uncontrolled diabetic for several years. Patients with diabetic ketoacidosis have elevated levels of available serum iron, likely due to release of iron from binding proteins in the presence of acidosis, elevated concentrations of glucose and iron enhanced surface GRP78 expression and resulting penetration through and damage of endothelial cells Mucorales in a receptor-dependent manner [4]. Authors have reported diabetes mellitus as a predisposing factor for mucormycosis in (36%–88%) of cases [8].

The symptoms and evolution of disseminated mucormycosis vary widely, reflecting the host as well as the location and degree of vascular invasion and tissue infarction in the affected organs. The variable presentation of disseminated mucormycosis requires a high index of suspicion to enable early diagnosis.

Patients with uncontrolled hyperglycemia, particularly those with ketoacidosis, are the most susceptible. [[9], [10], [11]].

Mucormycosis may be the first manifestation in some patients with undiagnosed diabetes mellitus [12], but it is rarely observed in those with metabolically controlled diabetes [13]. Type 1, type 2, and secondary diabetes mellitus are all reportedly risk factors for mucormycosis. Roden et al. [8] showed that diabetic patients represented 36% of 929 reported cases, but there was a decreased incidence of mucormycosis in diabetics over time.

Pulmonary mucormycosis is high (76%); it is even higher in severely immunosuppressed patients [14]. May develop as a result of inhalation or by hematogenous or lymphatic spread [15]. Pulmonary mucormycosis clinical presentation is non-specific. Patients may present with an unremitting high-grade fever (38 °C), that is unresponsive to broad-spectrum antibiotics and non-productive cough is a common presenting symptom. Hemoptysis and pleuritic chest pain are uncommon symptoms.

In rare circumstances, pulmonary mucormycosis can present as an endobronchial or tracheal lesion, especially in diabetics. Endobronchial mucormycosis can cause airway obstruction, resulting in lung collapse, and can lead to invasion of hilar blood vessels with subsequent massive hemoptysis [[16], [17], [18]].

The most frequent findings include infiltration, consolidation, nodules, cavitation’s, atelectasis, effusion, posterior tracheal band thickening, hilar or mediastinal lymphadenopathy, and even normal findings [19,20]. A study of 32 cases demonstrated the most common radiologic findings of pulmonary mucormycosis as consolidation (66%), cavitation more than 40%, pleural effusions (28%) [6]. Also there is a sign called a reversed halo sign, a focal round area of ground glass attenuation surrounded by a ring of consolidation, is more common in patients with mucormycosis than in those with other invasive pulmonary fungal infections [21].

Although our patient was having atypical GI symptoms, his histopathology was showing no fungal organism, we think that the inflammatory process in the spleen spread locally and invaded the fundus of the stomach.

Gastrointestinal mucormycosis is uncommon and seldom diagnosed in living patients. In such cases, diagnosis is delayed, and the mortality rate is as high as 85% [8]. Gastrointestinal mucormycosis is acquired by ingestion of pathogens in foods such as fermented milk and dried bread products. Consumption of fermented porridges and alcoholic drinks derived from corn may promote gastric mucormycosis.

Gastrointestinal mucormycosis can occur in any part of the alimentary system, but the stomach is most commonly affected, followed by the colon and ileum [[22], [23], [24]].

The diagnosis of gastrointestinal mucormycosis is usually delayed, because its nonspecific presentation requires a high degree of suspicion, usually made by biopsy of the suspected area during surgery, endoscopy or by culturing gastric aspirates. The infection usually presents with an appendicular, cercal, or ileac mass or gastric perforation that may be associated with frequently massive upper gastrointestinal tract bleeding [[25], [26], [27]]. In premature neonates, gastrointestinal mucormycosis presents as necrotizing enterocolitis, whereas in neutropenic patients, it does so as a mass like appendicular or ileal lesion [28,29]. Neutropenic fever, typhlitis, and hematochezia also can occur in neutropenic patients.

Gastrointestinal mucormycosis can also involve the liver, spleen, and pancreas. The fungus can invade bowel walls and blood vessels, resulting in bowel perforation, peritonitis, sepsis, and massive gastrointestinal hemorrhage, which is the most common cause of death [[26], [27], [28], [29]].

Antifungal therapy alone is insufficient for mucormycosis, and surgical debridement for all infected tissue is often required. Aside from the resistance of some fungal strains to amphotericin B, the pathogenesis of mucormycosis including angioinvasion, thrombosis, and tissue necrosis result in poor penetration of anti-infective agents to the site of infection [7]. Surgical debridement of infected and necrotic tissue should be performed immediately. Delayed therapy for 3 months resulted in a 2-time increase in mortality [5]. Review of 929 reported cases demonstrates that antifungal therapy and surgery are independently associated with a decreased risk of mortality [8].

## Conflicts of interest

All of authors have no conflict of interest.

## Sources of funding

This research did not receive any specific grant from funding agencies in the public, commercial, or not-for-profit sectors.

## Ethical approval

In our institute, ethical approval is exempted, depend on acquired patient consent.

## Consent

Written informed consent was obtained from the patient for publication of this case report. A copy of the written consent is available for review by the Editor-in-Chief of this journal.

## Author contributions

All authors contributed to manuscript preparation, manuscript editing, manuscript review.

## Registration of research studies

We don’t need to register this work.

## Guarantor

The Guarantor is DR. Ashwag Alharbi.

## Provenance and peer review

Not commissioned, externally peer reviewed.
